# Resveratrol Reverses Thioacetamide-Induced Renal Assault with respect to Oxidative Stress, Renal Function, DNA Damage, and Cytokine Release in Wistar Rats

**DOI:** 10.1155/2019/1702959

**Published:** 2019-09-10

**Authors:** Seema Zargar, Mona Alonazi, Humaira Rizwana, Tanveer A. Wani

**Affiliations:** ^1^Department of Biochemistry, College of Science, King Saud University, P.O. Box 22452 Riyadh 11451, Saudi Arabia; ^2^Department of Microbiology, College of Science, King Saud University, P.O. Box 22452 Riyadh 11451, Saudi Arabia; ^3^Department of Pharmaceutical Chemistry, College of Pharmacy, King Saud University, P.O. Box 2457, Riyadh 11451, Saudi Arabia

## Abstract

**Background:**

Thioacetamide (TAA), a class 2B-type carcinogen, is a potent toxicant. Toxicities caused by this compound in various tissues due to oxidative stress, increase of proinflammatory markers, and apoptosis have been reported; however, reports on kidney toxicity are negligible. Resveratrol (RSV), on the other hand, has demonstrated antioxidant and anti-inflammatory effects in different cases. Resveratrol's protective effects against TAA kidney toxicity were investigated in four rat groups.

**Methodology:**

Four groups of rats were studied as follows (*n* = 8): control group, where rats were fed normal diet and water; TAA group, where rats received 0.3% TAA in water for two weeks; RSV group, where rats received 10 mg/kg body weight (bw) of RSV as oral suspension for two weeks; and treated group, where rats orally received 10 mg/kg bw RSV and simultaneously received 0.3% TAA for two weeks. Kidney homogenates from all groups were analyzed for cytokine release (IL-4, TNF-*α*, and IFN-*γ*) and oxidative stress (lipid peroxidation, catalase, and 8-OHdG). The serum of rats was analyzed for the quantification of renal function markers (blood urea nitrogen (BUN), creatinine, and creatine kinase).

**Result:**

A significant increase in the renal function markers (BUN, 240%; creatinine, 187%; and creatine kinase, 117%), oxidative stress parameters (lipid peroxidation, 192% increase; catalase, 30.5% decrease), cytokines (IL-4, 120%; TNF-*α*, 129%; and IFN-*γ*, 133%), and DNA damage was observed in the TAA-treated group. All changes were significantly reversed in the group treated with RSV and TAA (*P* < 0.05) in combination, with no significant difference compared to the control group.

**Conclusion:**

We conclude that resveratrol shows protection against TAA toxicity in rat kidney with respect to DNA damage, oxidative stress, renal function and cytokine release.

## 1. Introduction

Thioacetamide (TAA; CH_3_CSNH_2_), an organosulfur compound, is commonly used as a fungicide [1, 2] owing to its generation of sulfide ions that prevent the germination of fungal spores. TAA is also widely used as an *in situ* source of sulfide ions in qualitative inorganic analysis to replace hydrogen sulfide in the pharmaceutical and chemical industries [3, 4]. The routes of human exposure to TAA include the generation of toxic fumes inhaled/ingested or absorbed through the skin. TAA is a model toxicant of choice due to its water-soluble nature and remarkable ability to induce assault [5]. TAA belongs to the class 2B-type carcinogens and results in acute liver and cytomegaly [6]. Acute exposure to TAA leads to necrosis as well as changes in chronic calcium permeability to the membrane due to an imbalance in calcium uptake, leading to apoptosis in the liver tissue [6–8]. TAA affects the ending of the proximal renal tubule by causing cell death [9]. When TAA is bioactivated, thioacetamide S-oxide is formed which leads to the generation of peroxide radicals further leading to the generation of reactive oxygen species (ROS) [1]. ROS initiates oxidation reactions such as lipid peroxidation to unsaturated lipids or triggers other reactions with sulfhydryl compounds, leading to liver injury [6, 10–12]. The metabolites generated are later distributed among several organs including the liver, kidney, adrenals, bone marrow, plasma, and other tissues [13], hence can modify amine lipids and proteins leading to further systemic oxidative stress, cytokine release, and altered kidney function that remain poorly understood. Resveratrol (RSV) (3,5,4′-trihydroxy-trans-stilbene), a natural polyphenolic compound found in grapes, berries, and many other plant species, is well known for its antioxidant properties [14]. RSV has demonstrated its protective activity against many oxidative stresses and inflammation [15, 16]. In addition, it has exhibited many health benefits including antioxidant [17], antimutagenic [18], anti-inflammatory [19], estrogenic [20], antiplatelet [21], anticancer [22], and cardioprotective [23] properties.

In the present study, we administered RSV- to TAA-treated rats to examine its effect on the levels of cytokine release, oxidative stress, and kidney function.

## 2. Materials and Methods

All chemicals required in this study including TAA were from Sigma-Aldrich (St. Louis, MO, USA) and RSV from EMD Millipore (Calbiochem, Billerica, MA, USA).

### 2.1. Experimental Protocol

32 male Wistar rats (4 weeks old; 70-80 g) were randomly divided into four different groups with eight rats each. The groups were categorized as control group; TAA group, rats receiving TAA; RSV group, rats receiving RSV; and TAA+RSV group, rats simultaneously receiving RSV and TAA.

TAA dosage was based on a previous literature [12]. Since RSV is insoluble in water, the suspension of 10 mg per mL stock was prepared and 10 mg/kg bw of the stock administered to rats by oral gavage.

All groups were sacrificed by carbon dioxide asphyxiation. The study was approved by the institutional review board for animal ethics (protocol no. 6828/2017), and every attempt was made to follow the guidelines. The control group was fed standard laboratory chow and water for two weeks, while in the TAA group, rats drank water containing 0.3% for two weeks. For the RSV group, rats were given 10 mg/kg/body weight (bw) of RSV as an oral suspension (the suspension was prepared as 10 mg/mL in water) for two weeks; rats in the TAA+RSV group were orally given a simultaneous 10 mg/kg bw RSV suspension with 0.3% TAA in water for 2 weeks.

### 2.2. Sample Preparation

Blood was drawn from the tail vein and the serum extracted and stored in a -80°C freezer for future use. The kidneys were dissected, washed, weighed, homogenized, and sonicated in normal saline using an ultrasonic cell disrupter from Vibra cell 72434 (Bioblock, Illkrich Cedex) [24]. All homogenates were centrifuged at 4,000 rpm for 5 min at 4°C. The resulting suspension was sonicated four times and stored in a -70°C freezer after centrifuging at 5000 rpm for 6 min at 4°C. Prior to performing the assay, all samples were diluted to 0.01 mol/L in PBS.

### 2.3. Assay of Cytokines (TNF-*α*, IL-4, and IFN-*γ*)

TNF-*α* was measured using the Sea-133ra ELISA kit from Cloud-Clone Corporation (CCC, USA) according to the method of Zargar et al. [11]. A precoated 96-well microplate with polyclonal antibody specific to TNF-*α* was added to the samples and standards. The unbound solution was removed via several rounds of washing. Avidin-conjugated horseradish peroxidase (HRP) was added and the plate washed. TMB substrate was added for the development of enzyme color and the reaction terminated by the addition of sulfuric acid. The change in color was measured at 450 nm. Each sample was measured in duplicate and the level of TNF-*α* determined by comparing the optical density (OD) of samples using the standard curve. Concentration of TNF-*α* was presented as pg/100 mg protein.

IL-4 and IFN-*γ* were measured using an ELISA kit from Cloud-Clone Corporation. Briefly, the 96-well microplates were pre-coated with the respective polyclonal antibody. The kit standards and samples were added according to the manufacturer's instructions and the unbound solutions removed via washing. Avidin-conjugated horseradish peroxidase (HRP) was later added to the plate. The unbound material was washed after the addition of the substrate solution and the reaction terminated by adding sulfuric acid. The change in absorbance was measured at 450 nm. Each sample was measured in duplicate and the concentrations of IL-4 were determined by comparing the OD of samples using a standard curve. Concentration of IL-4 was presented as pg/100 mg protein. For IFN-*γ* measurement, IFN-*γ* precoated antibody plates were treated with kit standards and the homogenates incubated for 1 h at 37°C. Following the addition of detection reagent, the plate was again incubated at 37°C for 1 h. After washing, the detection reagent B was added and the plate incubated for 30 min. Finally, the substrate solution was added, and after a 20 min incubation, the stop solution was added to terminate the reaction; absorbance was then recorded at 450 nm. Each sample was measured in duplicate and the level of IFN-*γ* presented in pg/100 mg of protein after comparison to the standard curve.

### 2.4. Assay of DNA Damage and Oxidative Stress

To examine DNA damage in cells, we assessed 8-hydroxy-2-deoxyguanosine (8-OHdG), a global marker of oxidative stress. The 8-OHdG was measured using an ELISA kit from Abnova Corporation, Taipei City (Taiwan). Briefly, 50 *μ*L of homogenate followed by 50 *μ*L of the detection solution was added to each precoated well and the plate incubated at 37°C for 1 h. The plate was then washed several times and inverted on dry tissue towels. A volume of 100 *μ*L of the working solution, reagent B (detection reagent), was added to each well. The plate was then incubated for 45 min at 37°C and washed five times. A volume of 90 *μ*L of substrate was later added to each well. The plate was incubated again at 37°C in the dark for 15-30 min. Finally, 50 *μ*L of the stop solution was added and the change in color determined at 450 nm. The level of 8-OHdG was determined in duplicate against a standard curve using a four-parameter logistic (4-PL) curve-fit software. The 8-OHdG was presented as ng/100 mg of DNA.

For lipid peroxide formation, 1.5 mL of 20% trichloroacetic acid was added to the preincubated tissue homogenates and centrifuged at 600 *g* for 10 min at 4°C. Further, 0.67% of thiobarbituric acid was added and the reaction mixture boiled for 15 min. Absorbance was recorded upon cooling at 535 nm using a blank reagent [25].

For catalase (CAT) activity, the homogenate was added to produce a final volume of 1.8 mL with sodium phosphate buffer (0.4 M; pH 7.2). At a later time, 1.2 mL of H_2_O_2_ was added to initiate the reaction and change in absorbance recorded at 240 nm for 2 min. One unit of CAT represents the amount in *μ*moles of H_2_O_2_ decomposed in 1 min with 43.6 M^−1^ cm^−1^ as the molar absorbance of H_2_O_2_ [26].

Total protein levels from homogenates were measured using bovine serum albumin as the standard [27]. The protein amount was calculated from a standard curve. Protein values are expressed as mg/g of fresh tissue.

### 2.5. Assay of Kidney Function Markers

The following parameters were measured using colorimetric kits from Human (Diagnostics Worldwide) (Wiesbaden, Germany): blood urea nitrogen (BUN), serum creatinine, and creatine kinase. To measure BUN, 10 *μ*L serum was added to 1 mL of the working reagent and the samples were incubated at 37°C for 30 s; absorbance was immediately recorded at 340 nm and after 1 min to determine the serum urea concentration. The BUN concentration was later determined by dividing serum urea concentration with the conversion factor 2.14, evaluated based on molecular weights of both BUN and urea. For serum creatinine and creatine kinase estimation, 10 *μ*L serum was added to 1 mL of the working reagent and incubated at 37°C for 30 s.

The absorbance of creatinine was immediately recorded at 500 nm and after 2 min; change in absorbance was directly proportional to creatinine in the sample. The absorbance of creatine kinase was recorded at 450 nm.

Histological analysis of the middle third part of nonclipped kidney was performed by modern enclosed tissue processor (Leica Biosystems, US); sections of 5–6 *μ*m thickness were stained with hematoxylin-eosin (HE). Images of at least five randomly selected areas of each sample were photographed at 40x magnification and analyzed for kidney injury by an expert pathologist who was blinded to the sample assignment in the experiment.

### 2.6. Statistical Analysis

All results are expressed as mean ± SD. The data statistically represented by number, mean, and SD were recorded. Comparison of different groups was performed using one-way ANOVA with Tukey's multiple comparison test. Comparison of all treatment groups was performed against the control group. A probability value (*P* value) ≤0.05 was deemed significant. All statistical calculations were performed using SPSS program (Statistical Package for Social Science version 11.0).

## 3. Results

**Figure 1:**
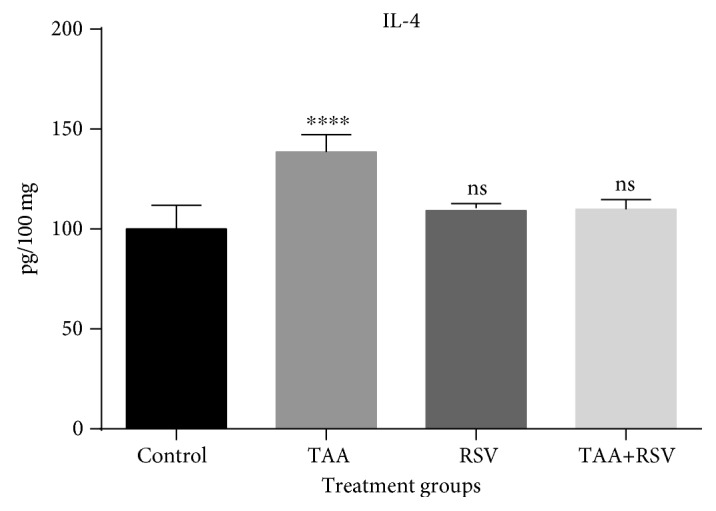
Effect of thioacetamide and resveratrol on the IL-4. IL-4 concentrations are expressed as pg/mg protein (*n* = 8) for the treated groups compared to the control group. ^∗∗∗∗^*P* < 0.0001; ns: nonsignificant.

**Figure 2:**
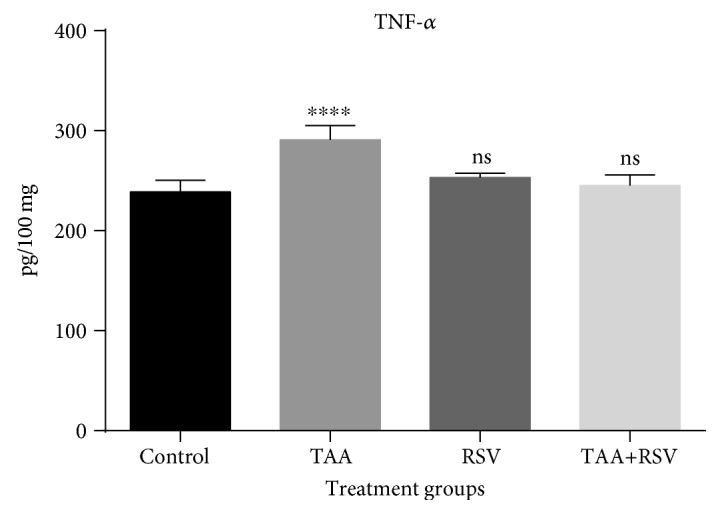
Effect of thioacetamide and resveratrol on TNF-*α*. TNF-*α* concentrations are expressed as pg/mg protein (*n* = 8) for the treated groups compared to the control group. ^∗∗∗∗^*P* < 0.0001; ns: nonsignificant.

**Figure 3:**
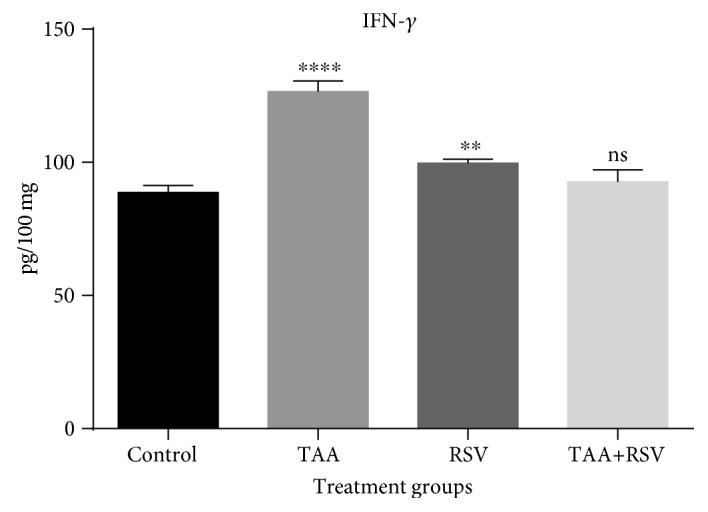
Effect of thioacetamide and resveratrol on IFN-*γ*. IFN-*γ* concentrations are expressed as pg/mg protein (*n* = 8) when treated groups are compared to the control group. ^∗∗∗∗^*P* < 0.0001; ^∗∗^*P* < 0.01; ns: nonsignificant.

The TAA group that received 0.3% TAA dissolved in water for two weeks experienced a significant increase in IL-4. Rats administered with a simultaneous treatment of the same amount of TAA and 10 mg/kg/bw of RSV dissolved in water for two weeks experienced a significant decrease when compared to the TAA-treated group (*P* < 0.05) (Figure 1). TNF-*α* was also significantly increased with TAA; however, a significant reversal occurred in the rat group that received simultaneous treatment of TAA and RSV (*P* < 0.05). A significant difference was not found when the RSV group was compared to the control group (Figure 2). Similarly, IFN-*γ* levels were significantly increased by TAA treatment and this effect was reversed by simultaneous RSV treatment with TAA. RSV treatment also resulted in a significant increase in IFN-*γ* levels when compared to the control group (*P* < 0.05) (Figure 3).

**Figure 4:**
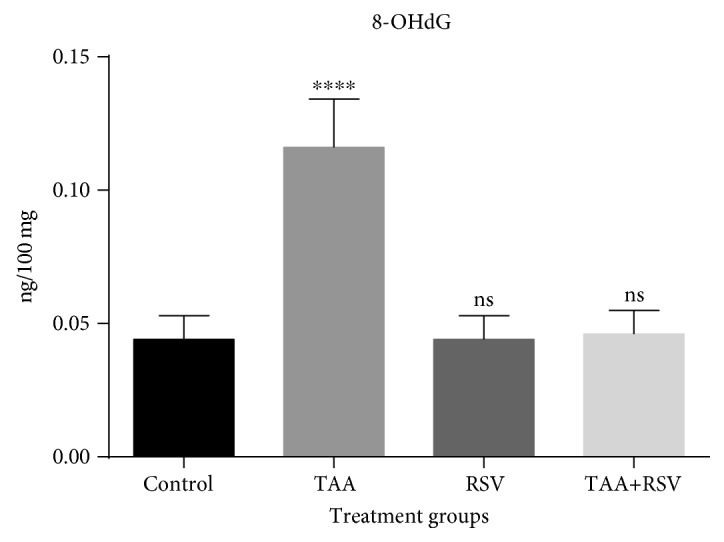
Effect of thioacetamide and resveratrol on 8-OHdG. 8-OHdG concentrations (*n* = 8) are expressed as ng/100 mg tissue when treated groups are compared to control group. ^∗∗∗∗^*P* < 0.0001; ns: nonsignificant.

The marker, 8-OHdG, is considered ideal for genotoxicity. The genotoxic effect in cells was increased by TAA and the increased levels of 8-OHdG significantly reduced in the rat group simultaneously treated with RSV (*P* < 0.05) (Figure 4). RSV treatment did not exhibit any significant difference with respect to the control group. Lipid peroxidation was also significantly increased by TAA treatment while RSV treatment significantly decreased the level of lipid peroxides. When both were simultaneously used, the increase caused by TAA was significantly and completely reversed (*P* < 0.05). Catalase was also significantly decreased by TAA treatment; however, combined therapy with RSV completely reversed the altered levels (*P* < 0.05) (Table 1). Separately and combined, TAA and RSV also altered the biochemical markers of kidney function (Table 1). BUN was significantly increased with TAA treatment while RSV treatment resulted in no significant alteration in the BUN level. When both the TAA and RSV drinks were simultaneously ingested, the increase in BUN caused by TAA ingestion was completely reversed (*P* < 0.05). Creatinine levels were significantly increased by TAA treatment while in the combination therapy, TAA and RSV treatment significantly reversed the increased level compared to the TAA-treated group. Creatine kinase levels were also significantly increased by TAA treatment when compared to control; RSV treatment had no effect on creatine kinase concentration. When both RSV and TAA were simultaneously ingested, the levels of creatine kinase were significantly reversed to normal (*P* < 0.05).

**Figure 5:**
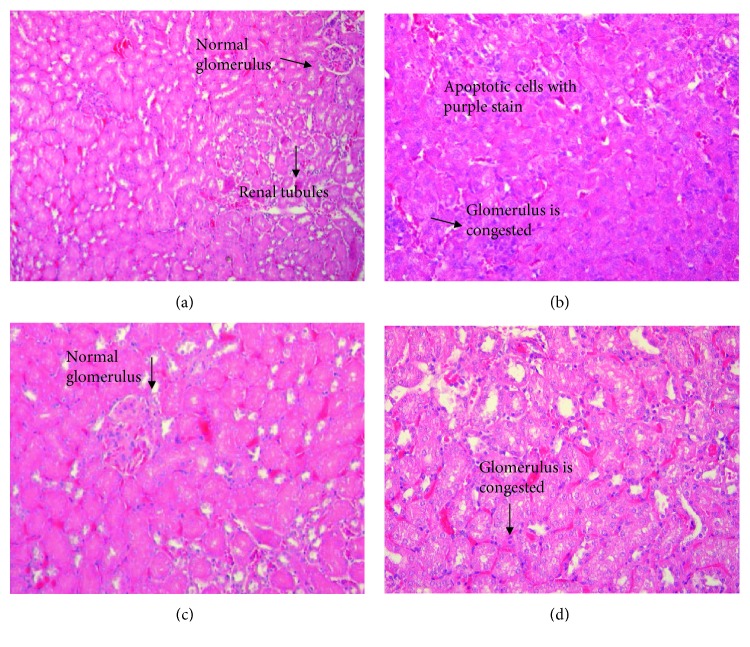
HE staining of thioacetamide and resveratrol treatments in kidney tissue sections at 40x. (a) Control group: having normal glomerulus and kidney tubules. (b) TAA-treated group: TAA treatment led to distortion of the glomerulus with apoptotic cells. (c) RSV-treated group: having normal glomerulus and tubules. (d) RSV+TAA-treated group: treatment with RSV before TAA preserved glomerular congestion while congestion of tubules and apoptotic cells were completely treated.

TAA insult led to a distortion in the glomerulus with significant congestion; RSV treatment, however, preserved the glomerular structure. Treatment with RSV before TAA preserved glomerular structure but the congestion of tubules could not be protected (Figure 5).

## 4. Discussion

TAA, an organosulfur fungicide, is a famous carcinogen that causes centrilobular hepatic necrosis in rats. RSV has been demonstrated to protect against the occurrence of many diseases such as diabetes, coronary heart diseases, tumor and obesity, and some oxidative stresses [28, 29]. In this study, RSV was demonstrated to exert protective effects against TAA-induced assault with respect to oxidative stress, renal function, DNA damage, and cytokine release on kidney tissue.

Renal function markers (BUN, creatinine, and creatine kinase) and the free radical scavenging (lipid peroxidation, catalase, and 8-OHdG) protection of RSV were assessed along with the relevant cytokine markers (IL-4, TNF-*α*, and IFN-*γ*). An increase in BUN, creatinine, and creatine kinase in the TAA-treated group indicated insufficiency in renal function. Previous studies reported that in acute tubular necrosis, tubular injury is mainly responsible for the reduced glomerular filtration. It was also suggested that the tubular abnormalities involved are blockage of tubules causing backward flow of glomerular filtrate [30]. Thus, renal insufficiency in TAA-treated rats might be secondary to ROS [31]. Reduced catalase activity was observed in the TAA group (Table 1) when compared to the control group, suggesting a decreased antioxidant potential in the TAA group. In addition, the catalase levels observed in the RSV- and TAA-treated group were similar to that of the control group, suggesting a reversal of the reduced antioxidant activity due to RSV treatment. It was found that the inflammatory markers, IL-4, TNF-*α*, and IFN-*γ*, were significantly reversed in the rat group simultaneously treated with TAA and RSV when compared to the increased levels observed in the TAA-treated group. Oxidative stress in other tissues caused by TAA has been reported in many studies; however, studies on kidney tissue are almost negligible [1, 32–34]. Many reports suggest that the oxidative stress caused by TAA may cause many diseases and pathologies. In this study, we found an increase in IL-4 expression in the TAA-treated kidney of rats, which was completely reversed by RSV treatment. Oxidative stress is an essential trigger for the activation of NF*κ*B in ischemia reperfusion injury. This results in the activation of p38 MAPK, which may be involved in the NF*κ*B activation that leads to TNF-*α* production [35]. Other studies have reported the induction of IL-6 expression during the development of acute kidney injury both in humans [36] and in experimental animal models [37, 38]; however, a study of IL-4 expression was not performed. IL-4 is a cytokine that can affect the activity of many types of tissues and can possess both proinflammatory and anti-inflammatory properties. Its upregulation leads to the infiltration of neutrophils in the renal tissue, hence the progression of renal injury [39]. In this study, we found that TAA treatment increased IFN-*γ* levels with respect to the control (i.e., inflammation at selected dose (10 mg/kg bw)). The primary function of IFN-*γ* is to limit damage to tissues in inflammation that would activate macrophages and NK cells to name a few that play major roles in tissue repair. Many studies have reported that IFN-*γ* induction increases ROS production in cells and increased ROS production contributes to IFN-*γ*-induced cell apoptosis to serve as a preventive measure in tissue injury [40, 41].

TAA increased DNA damage with increased 8-OHdG generation; however, treatment with RSV reversed this change. 8-OHdG is one of the markers induced by ROS that reflect oxidative damage [42]. Increased levels of 8-OHdG in cells is the best indicator of oxidative stress caused by degenerative diseases such as cancer [43]. Consistently, Zhang et al. [44] found induced level of 8-OHdG in urine sample from subjects exposed to formaldehyde. In addition, increased levels of 8-OHdG due to oxidative DNA damage based on urine samples of children with acute leukemia have been reported [45]. Increase in lipid peroxidation has been reported in many oxidative stress-induced renal injuries [46–48]. In our study, TAA also caused an increase in lipid peroxidation, and the effect was reversed in the group simultaneously treated with RSV and TAA. We emphasize its simultaneous use with TAA exposure, if any.

## Figures and Tables

**Table 1:** Effect of thioacetamide-induced changes alone and combined with resveratrol on the levels of kidney function and oxidative stress markers. Data are represented as mean ± SD of three independently performed experiments; each group had 8 rats for all experiments conducted: TAA-treated, 0.3% for two weeks; RSV-treated, 10 mg/kg bw for 2 weeks; and TAA+RSV-treated, 0.3%+10 mg/kg bw simultaneously for 2 weeks.

Parameters	Control	TAA-treated	RSV-treated	TAA+RSV-treated
BUN (mg/mg protein)	18.31 ± 2.9^b^	44.03 ± 1.9^acd^	20.7 ± 1.6^b^	17.88 ± 2.0^b^
Creatinine (mg/mg protein)	0.31 ± 0.06^bcd^	0.58 ± 0.03^ac^	0.44 ± 0.02^bd^	0.37 ± 0.03^bc^
Creatine kinase (U/mg protein)	57.71 ± 2.6^b^	67.66 ± 1.6^ac^	58.26 ± 1.8^b^	60.28 ± 5.9^b^
Lipid peroxidation (mmoles/mg protein)	2.20 ± 0.537^bc^	4.24 ± 0.57^acd^	1.81 ± 0.71^abd^	2.38 ± 0.12^b^
Catalase (U/mg protein)	1.08 ± 0.46^bd^	0.33 ± 0.06^ad^	0.98 ± 0.22^b^	1.33 ± 0.23^b^

^a^Significant (*P* < 0.05) compared to control; ^b^significant (*P* < 0.05) compared to TAA-treated group; ^c^significant (*P* < 0.05) compared to with the RSV-treated group; ^d^significant (*P* < 0.05) compared to simultaneous treatment of TAA+RSV-treated group.

## Data Availability

The data used to support the findings of this study are available from the corresponding author upon request.
